# Procedural Complications of Spinal Anaesthesia in the Obese Patient

**DOI:** 10.1155/2012/165267

**Published:** 2012-07-30

**Authors:** Manuel Wenk, Christian Weiss, Michael Möllmann, Daniel Matthias Pöpping

**Affiliations:** ^1^Department of Anesthesiology, Intensive Care and Pain Medicine, University Hospital Muenster, Albert-Schweitzer Campus 1, A1, 48149 Muenster, Germany; ^2^Medical School, University of Muenster, 48149 Muenster, Germany; ^3^Department of Anesthesiology and Intensive Care, St. Franziskus Hospital, 48145 Muenster, Germany

## Abstract

*Background*. Complications of spinal anaesthesia (SpA) range between 1 and 17%. Habitus and operator experience may play a pivotal role, but only sparse data is available to substantiate this claim. *Methods*. 161 patients were prospectively enrolled. Data such as spread of block, duration of puncture, number of trials, any complication, operator experience, haemodynamic parameters, was recorded and anatomical patient habitus assessed. *Results*. Data from 154 patients were analyzed. Success rate of SpA in the group of young trainees was 72% versus 100% in the group of consultants. Trainees succeeded in patients with a normal habitus in 83.3% of cases versus 41.3% when patients had a difficult anatomy (*P* = 0.02). SpA in obese patients (BMI ≥ 32) was associated with a significantly longer duration of puncture, an increased failure ratio when performed by trainees (almost 50%), and an increased number of bloody punctures. *Discussion*. Habitus plays a pivotal role for SpA efficiency. In patients with obscured landmarks, failure ratio in unexperienced operators is high. Hence, patient prescreening as well as adequate choice of operators may be beneficial for the success rate of SpA and contribute to less complications and better patient and trainee satisfaction.

## 1. Background

Ever since the introduction of spinal anaesthesia more than a century ago, complications have been part of the technique; failed or insufficient block, headaches, nausea, vomiting, and pain around the injection site are common minor complications [1, 2]. The technique of spinal anaesthesia (SpA) is considered a basic skill, however, one that first has to be mastered. According to literature, the incidence of failed or partially failed SpA ranges between 0.5 and 17% [3–5]. The incidence of postdural puncture headaches (PDPHs) ranges between 0,7 and 11% based on the type of needle used [6, 7], and transient neurologic syndromes can still be observed after SpA with an incidence of 0–7% [8].

As with many other procedures in medicine, intuition suggests that procedure-specific experience of the operator should be beneficial and reduce complications. However, there is only sparse data available to demonstrate that this is the case for SpA [9, 10]. 

Furthermore, with an increasing number of severely obese patients in western society, anesthesiologists are—more than ever—faced with patients where the individual habitus causes a challenge to perform a seemingly simple basic skill like SpA because it relies on identifiable anatomical structures termed “landmarks.” These can be completely obscured in the obese patient [11, 12].

The aim of this study was to evaluate the impact of the individual patient habitus on the success rate of SpA and the incidence of immediate complications related to SpA in the context of operator experience. 

## 2. Methods

After approval from the Ethics Committee of the Medical Faculty of the University of Muenster (protocol 2009-459-f-S), 161 patients planned for elective orthopedic or vascular surgical procedures of the lower limb under SpA were enrolled in the study. Informed consent was obtained from each patient. 

Operators were divided into two groups (*n* = 5/each group). Group T were anesthetic trainees with ≤1 year experience in anaesthesia and group C anesthetists with ≥5-year experience in anaesthesia and >150 previously performed SpA as well as ongoing regular exposure to SpA.

Exclusion criteria were as follows:

- <18 or >90 years of age,
- coagulation disorders or any combination of the following: INR > 1.5, aPTT > 40 sec, thrombocytes < 100.000 per microliter blood,
- neurological disorders,
- sepsis or severe infection or local infection around the typical injection site,
- known allergies to local anesthetics, and
- American Society of Anesthesiologists Score (ASA) ≥ IV.

Besides demographic data, we recorded the following characteristics: number of puncture trials, change of spinal segment, bleeding from the introducer or the spinal needle, duration of procedure, paresthesias during puncture, spread of sensory and motor block, failed or partially failed SpA as well as hemodynamic changes in blood pressure and heart rate. A relevant hypotensive episode was defined as a systolic blood pressure <85 mmHg or a decrease in systolic pressure >30% below the initial systolic blood pressure.

An anaesthesiologist with >20-year experience as an anesthetic consultant assessed each patients' spinal anatomy based on palpation as well as X-rays, when available. Patients were divided into an “easy” and a “difficult habitus for SpA” group. The habitus was considered “difficult” when no spinous processes were palpable at the L3–L5 level and above, which could be used as landmarks to guide the operator to identify a midline. Furthermore, in patients with lumbar scoliosis and subsequent longitudinal rotation of the spinous processes towards the concave side as identified by X-ray, the habitus was considered “difficult.”

All patients were attached to standard monitoring (noninvasive blood pressure, electrocardiogram, and peripheral oxygen saturation). An intravenous access was established, and an infusion of 1000 mL of a balanced electrolyte solution (Sterofundin-ISO, B.Braun, Melsungen, Germany) was started. Patients were then turned into a lateral position, and after usual sterile preparations SpA was performed with a 25-gauge pencil point spinal needle (PenPoint, B.Braun, Melsungen, Germany). A standard introducer needle was used to facilitate spinal needle puncture. Once a free flow of cerebrospinal fluid (CSF) was obtained, the color of CSF was compared against a color scale measuring the amount of blood in CSF.

Local anesthetics used were isobaric bupivacaine 0.5% (3 mL) for endoprosthetic surgery or isobaric ropivacaine 0.5% (2.5–4 mL) for all other procedures. If the surgical procedure was expected to be of longer duration, 0.1 mg morphine was additionally injected into the subarachnoid space.

Statistical analysis was performed using SPSS Statistics 18.0 (SPSS Inc., Chicago, IL, USA). Categorical variables are expressed as frequency and percentage, whereas continuous variables are represented as means with standard deviation or as median and interquartile range (25th percentile; 75th percentile). Before statistical testing, each continuous variable was analysed exploratively for its normal distribution using Kolmogorov-Smirnov test. The Mann-Whitney test was then applied for comparison of nonparametric variables between the two study groups. The nonparametric patients' baseline characteristics were assessed using the Kruskal-Wallis test. Friedman's signed rank test was used to compare the nonparametric time-dependent variables and the chi-square test for comparison of categorical variables.

Differences were considered as statistically significant at *P* < 0.05.

## 3. Results

161 patients were enrolled in the study. 7 patients were excluded due to changes in the treatment plan. Complete data sets of 154 patients were subsequently analyzed. 

Demographic data of all patients is displayed in Table 1.

Overall success rate of SpA in the group of young trainees was 72% versus 100% in the group of consultants. 51 (35%) patients were rated to have a “difficult” anatomy/habitus. Trainees succeeded to perform SpA in patients with an easy habitus in 83.3% of cases versus 52.4% when patients had a difficult anatomy (*P* = 0.005). When trainees failed a SpA, an operator from group C took over, and they were successful in 100% of the cases hence all patients enrolled in the study had the planned surgical procedure done under SpA.

Table 2 lists specific complications encountered in both operator groups and the two patient groups. Obese patients with a BMI ≥ 32 were significantly higher at risk to experience complications during SpA. Duration of puncture was longer, trainees failed SpA in almost half the cases, and there were significantly more bloody punctures and a higher incidence of paresthesias. Furthermore, even consultants required 3 or more punctures to perform successful SpA in 42.5% of the patients with a BMI ≥ 32.

The height of the achieved sensory and motor block was not related to weight or BMI of the patient.

Consultants caused less paraesthesias when performing SpA as compared to trainees; however, the difference was not statistically significant (*P* = 0.31). Patients that were rated to have a difficult habitus had significantly more paresthesias during puncture than patients with identifiable landmarks (13.2 versus 2%; *P* = 0.005). Furthermore, patients with a difficult habitus had significantly more pain during the procedure than patient with an easy habitus (11.3 versus 1.9%; *P* = 0.02).

Isobaric bupivacaine 0.5% in equipotent doses caused significantly more hypotensive episodes after intrathecal injection as compared to isobaric ropivacaine 0.5% (21 (25%) versus 2 (3.1%); *P* = 0.0002). There was no significant difference in hypotensive episodes between patients with a BMI < 30 versus ≥30 (*P* = 0.05). 

Bradycardia with a heart rate of 45 beats per minute or below was observed in 9 (6%) patients and was not significantly related to the local anaesthetic used but was significantly correlated with the level of puncture. 

Interestingly, patients who required 4 or more punctures to place a successful SpA had a significantly greater drop in blood pressure. 

On day one postoperatively, two patients (1.3%) showed typical features of a transient neurologic syndrome, 6 patients (3.9%) reported difficulties passing urine during the first 12 hours, but no patient required bladder catheterization. 15 patients (9.7%) had one or more episodes of PONV (Table 3).

No major complications such as severe hemodynamic disturbances, cardiac arrest, cauda equina syndrome, or permanent neurologic complications were observed. 

## 4. Discussion

Spinal anaesthesia has an excellent safety record in terms of major complications. However, there is a significant number of minor complications that—each on its own—may cause unpleasant sequelae for the patient [3, 4, 13]. The majority of complications are associated with the procedure itself. Insufficient or failed SpA ranges from 0 to 17% and bloody punctures as well as significant hypotension are not uncommon [3, 9]. The current study shows that the overall failure rate of SpA is comparable to previously published data. We have shown that success and failure rate appears to be directly dependent on the operator's experience and the individual patient habitus. Trainees failed significantly more attempts to perform SpA, had more difficulties placing SpA in patients with obscured landmarks, and had significantly more bloody punctures, and the procedure duration was significantly longer as compared to experienced specialists. It has been shown previously that SpA is a complex procedure that is more difficult to master than, for example, endotracheal intubation [14]. Furthermore, it has been estimated that the experience of around 100 performed SpA is required to achieve a 90% success rate [15]. Our data shows that young trainees had a success rate of 84% in patients with a normal anatomy, indicating that some trainees have probably mastered the technique while others were still on the ascending part of the learning curve. However, this picture changes completely when patients present with obscured landmarks or difficult anatomy. Trainees, who were able to perform SpA successfully in anatomically “easy” patients, suddenly faced a failure rate of 52% in those patients with a difficult habitus, significantly different to “easy” patients. Consultants were able to place a SpA even in the difficult patients but in 42.5% of cases, 3 or more punctures were required to position the spinal needle in the correct location. To our knowledge, this is the first study that specifically investigated the role of the individual patient habitus by rating landmarks and other anatomical features. Part of educating trainees is to accept that they do have a higher failure rate [16, 17], and it is the responsibility of the relevant societies to define what is an acceptable failure rate for which procedure [18]. Based on our findings we postulate that an experienced anesthesiologist should anatomically rate all patients who are about to receive SpA and if the habitus is considered to be difficult, young trainees should probably not perform SpA to avoid frustration and build a more solid foundation based on successfully performed punctures rather than failing every second attempt. However, from our data, it appears that young trainees do have a higher failure rate, but they do not cause significantly more complications. Hence exposure to the difficult patient is relatively safe, once a solid foundation of the technique has been established. We recommend that the level of supervision should be adequate to avoid that the operator's success or fail rate in these patients is significantly lower than in experienced operators. Multiple attempts by young trainees as well as experienced operators lead to a more significant reaction of hemodynamic parameters. Blood pressures dropped significantly more in patients where multiple attempts were necessary. We offer two possible explanations. Firstly, multiple attempts may lead to the operator changing spinal segments, and the direction is usually upwards thus causing more sympathetic block. Secondly, multiple attempts may cause stress and enhance anxiety in the patient hence causing disturbances of the autonomous sympathetic regulation. Last but not least, avoiding multiple attempts may also affect patient satisfaction, but we have not investigated that matter.

As a training tool for young trainees as well as a tool to use in the anatomically challenging patient, the introduction of ultrasound-guided SpA may be worthwhile to consider. Some studies have shown increased success rates when ultrasound is used in patients with obscured landmarks or difficult anatomy [19–21]. However, this might involve teaching both SpA and the use of an ultrasound machine to trainees at the same time, which may be an even bigger challenge. Furthermore, similar to current discussions on the comprehensive use of ultrasound for central venous catheter placement, it needs to be discussed whether trainees should in general learn to perform landmark techniques before they add ultrasound or vice versa.

Our study has limitations. Firstly, patients were not randomized to experienced or unexperienced operators but were consecutively allocated to operators available. Secondly, operators could not be blinded to patient habitus for obvious reasons. However, operators were blinded to the assessment of the anatomical structures and the subsequent grading. 

Since trainees were on the ascending part of the learning curve during the study period, repeated exposure to performing SpA itself may have influenced their individual performance and subsequently the results. Furthermore, our study was not powered to comment on incidences of rare major complications such as severe hemodynamic disturbances, cardiac arrest, cauda equina syndrome, or permanent neurologic complications since this was never the aim of this study.

## 5. Conclusion

Albeit a relatively safe technique, SpA has its problems and pitfalls, and our study has shown that increased operator-experience results in a higher success rate of SpA. Furthermore, the individual patient's habitus plays a pivotal role when trainees are involved in performing SpA. Even for experienced anesthesiologists this group of patients has its challenges, but the failure rate of SpA is still very low. We conclude that careful patient selection and prescreening as well as adequate choice of operators is beneficial for the success rate of SpA and may contribute to less complications, greater safety, better patient, and trainee satisfaction. 

## Figures and Tables

**Table 1:** Demographic data.

Age (years)	64.9 ± 15.0
Sex ratio (male/female)	64/90
Height (cm)	170 ± 9.7
Weight (kg)	79 ± 16.1
Body mass index (kg/m^2^)	27.2 ± 4.9
BMI ≤ 24.9 (*n*)	52
BMI ≤ 29.9 (*n*)	53
BMI ≤ 34.9 (*n*)	38
BMI ≤ 39.9 (*n*)	9
BMI ≥ 40 (*n*)	2
ASA I (*n*)	42
ASA II (*n*)	87
ASA III (*n*)	25

**Table 2:** Incidences of immediate complications for patients with “easy” and “difficult” habitus and the respective operator experience.

	Easy habitus			Difficult habitus		
	Trainees (*n* = 46)	Consultants (*n* = 57)	*P*	Trainees (*n* = 21)	Consultants (*n* = 30)	*P*
Duration of puncture (sec)	117 ± 80	63 ± 48	0.001^∗^	154 ± 89	102 ± 91	n.s.
Success on first puncture	19 (41.3%)	48 (84.1%)	0.002^∗^	2 (9.5%)	11 (36.7%)	0.03^∗^
Failed SpA	6 (13.0%)	0	0.01^∗^	10 (47.6%)	0	0.0003^∗^
Insufficient spread of SpA	2 (4.3%)	1 (1.6%)	n.s.	1 (4.8%)	1 (3.3%)	n.s.
Segment change	7 (15.2%)	3 (5.2%)	0.05	8 (38.1%)	12 (40%)	n.s.
Blood in introducer needle	5 (10.9%)	5 (8.8%)	n.s.	8 (38.1%)	9 (45%)	n.s.
Blood in CSF	4 (8.7%)	1 (1.6%)	n.s.	3 (14.3%)	5 (16.7%)	n.s.

Values indicate total number of patients and percentage. Column *P* displays the respective *P* values where ^∗^indicates significance (*P* < 0.05) and n.s.: not significant.

**Table 3:** Incidences of complications on day one postoperatively and respective operator experience.

Trainees	Consultants	*P*	Difficult anatomy	Easy anatomy	*P*	
Transient neurological syndrome (%)	2.2	1.1	n.s.	1.1	2.0	n.s.
Pain at insertion site (%)	4.4	9.5	n.s.	10.0	6.7	n.s.
Urinary retention (%)	0	0	n.s.	0	0	n.s.
PONV (%)	11.1	10.5	n.s.	14.0	8.9	n.s.

Values indicate total number of patients and percentage. n.s.: not significant.
